# Properties of Vulcanized Polyisoprene Rubber Composites Filled with Opalized White Tuff and Precipitated Silica

**DOI:** 10.1155/2014/913197

**Published:** 2014-02-06

**Authors:** Suzana Samaržija-Jovanović, Vojislav Jovanović, Gordana Marković, Ivana Zeković, Milena Marinović-Cincović

**Affiliations:** ^1^Faculty of Natural Science and Mathematics, University of Priština, Lole Ribara 29, 38220 Kosovska Mitrovica, Serbia; ^2^Tigar, Nikole Pašića 213, 18300 Pirot, Serbia; ^3^Institute of Nuclear Science Vinča, University of Belgrade, Mike Petrovića Alasa 12-14, 11000 Belgrade, Serbia

## Abstract

Opalized white tuff (OWT) with 40 **μ**m average particle size and 39.3 m^2^/g specific surface area has been introduced into polyisoprene rubber (NR). Their reinforcing effects were evaluated by comparisons with those from precipitated silica (PSi). The cure characteristic, apparent activation energy of cross-link (*E*_ac_) and reversion (*E*_ar_), and mechanical properties of a variety of composites based on these rubbers were studied. This was done using vulcanization techniques, mechanical testing, and scanning electron microscopy (SEM). The results showed that OWT can greatly improve the vulcanizing process by shortening the time of optimum cure (*t*_*c*90_) and the scorch time (*t*_*s*2_) of cross-linked rubber composites, which improves production efficiency and operational security. The rubber composites filled with 50 phr of OWT were found to have good mechanical and elastomeric properties. The tensile strengths of the NR/OWT composites are close to those of NR/PSi composites, but the tear strength and modulus are not as good as the corresponding properties of those containing precipitated silica. Morphology results revealed that the OWT is poorly dispersed in the rubber matrix. According to that, the lower interactions between OWT and polyisoprene rubber macromolecules are obtained, but similar mechanical properties of NR/OWT (100/50) rubber composites compared with NR/PSi (100/50) rubber composites are resulted.

## 1. Introduction

Modern engineering systems are being increasingly produced from components that combine two or more materials for enhanced performance. During the processing of rubber vulcanizates, mixing of filler and cross-linking are the two substantial parameters as the homogeneity of mixing and cross-linking significantly affect the properties of the vulcanizates. Recent investigations (from both a technological and fundamental point of view) show that the interfacial bonding strength has a profound influence on the failure of dissimilar or composite materials [1]. Silica has been used as nonblack reinforcing filler in the rubber industry for a long time. Reinforced rubber blends are suitable materials for industrial practice [2]. Obviously, in such systems both components (filler and rubber) have the reactive groups for the additional cross-linking reaction to take place [3]. Besides, the so-called “dispersion” forces there are a variety of other interactions between particles. A key one among them is hydrogen bonding, which tends to be a significant force in the case of fumed silica, where hydrogen bonding between surface silanol groups takes place. The surface of hydrated or precipitated silica is highly polar and hydrophilic because of the presence of numerous silanol groups [4, 5]. Many authors have investigated the role of filler networking in the elastic properties of elastomer composites [6–9].

In recent years, rubber composites have attracted great interest, both in industry and in academia, because they often exhibit remarkable improvements in materials properties when compared with the virgin polymer composites. Usuki et al. [10] prepared some EPDM/clay hybrids with montmorillonite, and the results showed that the tensile strength and storage modulus were improved and the permeability decreased 30% (compared with neat EPDM). Organo-montmorillonite (OMMT) as a substitute for carbon black in natural rubber compounds was researched by Arroyo et al. [11]. The mechanical properties of NR filled with 10 phr organoclay were comparable to those of the compound with 40 phr carbon black. Moreover, the organoclay improved the strength of the NR without any reduction in the elasticity of the material. Essawy and El-Nashar [12] studied the use of montmorillonite as a reinforcing and compatibilizing material for NBR/SBR rubber blend. Teh et al. [13] studied the effects of epoxidized natural rubber as a compatibilizer in melt compounded natural rubber/organoclay nanocomposites.

Similarly, the cure characteristics, clay dispersion, and thermomechanical properties of these nanocomposites were determined. Zheng et al. [14] studied the influence of clay modification on the structure and mechanical properties of EPDM/montmorillonite nanocomposites and showed that the OMMT layers were fully exfoliated in the EPDM matrix and the composites had good mechanical properties. Wang et al. [15] in researching the influence of fillers on free volume and gas barrier properties in SBR revealed that gas permeability is mainly influenced by fractional free volume and tortuous diffusional path effects attributed to the clay plate-like morphology. The properties of calcined and hydrous kaolin filled nylon 66 composites were investigated by Buggy et al. [16] with respect to particle size and surface treatment with an aminosilane coupling agent. Finally, Liang et al. [17] prepared isobutylene-isoprene rubber/organic modified clay nanocomposites by solution or melt intercalation, and the prepared nanocomposites exhibited outstanding mechanical properties and improved gas barrier properties.

Tuff usually named according to the nature of rock fragments, for example, bazalt's tuff, adenzit's tuff, rhyolitic tuff, and so forth. On the basis of glass and crystal contents in the tuff, they can be divided into vitroclastic and crystaloclastic. Some tuffs are composed mainly of glassy ash particles. Tuff belongs pyroclastic rocks which are hydrothermally altered with porphyritic texture. The color is from white to yellow white. It is easily crushed with sharp edges. Opalized tuff is volcanic material which suffered hydrothermal changes during geologic period.

In the present study, opalized white tuff (OWT) was used as the reinforcing agents in NR rubber compared to NR/precipitated silicate (PSi) composites and the curing, mechanical, heat aging resisting, and morphology properties of the corresponding rubber composites were analyzed.

## 2. Experimental

### 2.1. Materials

#### 2.1.1. Rubber

RSS 1 refers to ribbed smoked sheets (Malaysia), produced from natural rubber latex as ribbed sheets, by coagulation with acids and sheeting, properly air dried and smoked, and visually graded. Ribbed smoked sheets (RSS) are graded based on visual assessment of quality.

#### 2.1.2. Fillers

Opalized white tuff (OWT) with 40 *μ*m primary particle size and 39.3 m^2^/g specific surface area was obtained as an industrial product from the mining company AD Strmoš Probištip-Česinovo in FYR of Macedonia. It belongs to the pyroclastic rocks which are the products of explosive volcanic eruptionsis. The opalized tuff is constructed of the mineral tridymite, cristobalite, quartz, feldspat, and limonite. Vulkasil A1 (Bayer, Germany) (precipitated sodium aluminium silicate, namely, PSi) with a medium reinforcing effect was used. The chemical composition and physical properties of OWT and PSi are given in Table 1.

### 2.2. Methods of Preparations

Formulation of the composites is given in Table 2. The compounds (Table 2) were prepared using a laboratory mixing roll mill of dimensions 400 × 150 mm at a speed ratio of the rollers *n*_1_/*n*_2_ = 28/22, at a roller temperature of 40–50°C. The processing time after each component addition was about 2 min. The compound rubber was allowed to stand overnight before vulcanization. The rheometric characteristics were assessed by a Monsanto Oscillating Disc Rheometer R-100, according to the ASTM D2084-95 standard testing method. The optimum curing time (*t*_*c*90_) was determined at 160°C. The compounds were molded using an electrically heated hydraulic press (Indexpell, Kerala, India) under a pressure of 60 MPa at a temperature optimum curing time. These cured sheets were conditioned before testing (24 h maturation at 25°C).

### 2.3. Methods of Characterization

#### 2.3.1. Fourier Transform Infrared Spectra (FTIR-ATR) of Filler

Fourier transform infrared spectra (FTIR) were recorded on a Bruker IFS-66 spectrometer with an attenuated total reflection (ATR) attachment. The internal reflection element (IRE) chosen was a 45-degree KRS-5. Potassium bromide (KBr) used matrix material. The KBr pellets of samples were prepared by mixing (1.5–2.00) mg of samples, finely grounded, with 200 mg KBr (FT-IR grade) in a vibratory ball mixer for 20 s. The mixture is now transferred to a die that has a barrel diameter of 13 mm. This is then placed in a suitable press and pressed (evacuation is optional) at around 12,000 psi for one to two minutes. Re-crystallization of the KBr results in a clear glassy disk about 1 mm thick. This disk is now ready to be analyzed by transmission.

#### 2.3.2. Rheometric Characteristic

The cure characteristics: *M*_*l*_ (minimum torque), *M*_*h*_ (maximum torque), *t*_*c*90_ (optimum cure time), *t*_*s*2_ (scorch time), and CRI (cure rate index) were determined with a Monsanto Oscillating Disc Rheometer R-100 at 160°C in accordance with ASTM method D-2084.

#### 2.3.3. Cure Kinetics

The kinetic parameters for the cross-linking process, such as apparent activation energy of cross-link (*E*_ac_) and reversion (*E*_ar_) process, were calculated from the torque-time curves. The torque and time experiments were performed using an accelerated sulfur curing system with an oscillating disk rheometer (Monsanto Rheometer model 100C) at two temperatures: 180 and 190°C.

#### 2.3.4. Mechanical Properties

Mechanical properties, such as tensile strength, modulus (%), and elongation at break, were measured with a Zwick-1425 tensile tester according to the ASTM D412-98 standard testing method using a crosshead speed of 500 mm/min and at 25°C. For the tensile experiment, dumbbell samples were cut from a 2 mm thick molded sheet. The tensile properties of the blends were examined according to the ASTM D-412 standard testing method. Five samples from each formulation were tested. The hardness of the samples was measured, as per the standard ASTM D-2240 testing method. For hardness measurements, the sheets having an effective thickness of 6 mm were used. At least five measurements were recorded, and the average values were reported. To investigate the influence of thermal aging on the mechanical properties, the obtained reinforced elastomeric materials were performed in an air circulating oven operated at 100°C during 72 h and 168 h. The retained percentage values of tensile strength and elongation at break were calculated. After aging, hardness is given in point. The tensile properties (tensile strength and elongation at break) and hardness were measured before and after thermal aging has been studied.

#### 2.3.5. Microscopic Examination by SEM

Samples were immersed in liquid nitrogen for more than 15 min to cool down and then fractured immediately. The fractured surfaces of the blended materials were imaged by scanning electron microscopy (SEM) using a JEOL JSM-5400 model SEM. The samples were sputter coated with gold for 3 min under high vacuum with image magnifications of 3500x. The aim was to obtain some information on the mode of the fracture the condition of the matrix, and filler surfaces and dispersion.

## 3. Results and Discussion

### 3.1. Fourier Transform Infrared Spectroscopy of Fillers

**Figure 1:**
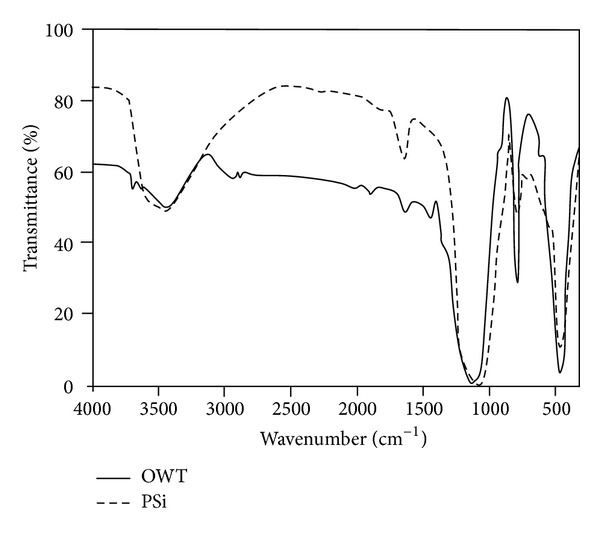
FTIR spectra of OWT and PSi.

The FTIR transmittance spectra of OWT and PSi are shown in Figure 1 and the assignment of the bands is shown in Table 3. The characteristic bands almost at 1119, 792, and 466 cm^−1^ correspond to the stretching, bending and out of plane of Si–O bonds [18, 19], respectively, for OWT, and 1088, 795, and 467 cm^−1^ for PSi. The position and the shape of the main Si–O vibration band at 1119 and 108 cm^−1^ show a stoichiometric silicon dioxide structure. Moreover some impurity vibration bands are seen in the FTIR spectra which were shown in Table 3; as it is observed from the spectra, these are too smaller than the main pick. The characteristic bands for all three types of SiO_2_ (quartz, tridimite, and cristoballite) are obtained at 1119 cm^−1^ for OWT and at 1088 cm^−1^ for PSi. The width of these bands dependents on the chaotic state of solids. The band at 907 cm^−1^ for OWT originated from stretching vibration of SiOH group. Its absence in PSi is a result of heat treatment during synthesizing process. A peak in the spectral range at around of 1635 and 1641 cm^−1^ is attributed to vibrations of –OH (molecular water) [20]. The FTIR spectra also showed a large amount of OH groups at around of 3438 and 3480 cm^−1^.

### 3.2. Rheometric Characteristics

For filled compounds, type and content of filler affect the cure characteristics [21]. Lots of functional groups such as hydroxyl, silanols, siloxane, and hydrogen bonded water exist on the silica surface but the amount is small.  Table 4 shows the rheometric characteristics, such as delta torque Δ*M* (difference between the maximum and minimum torques), scorch time (*t*_*s*2_), and optimum cure time (*t*_*c*90_), of the compounds at 160°C. Minimum torque (*M*_*l*_) is directly related to the viscosity of the compounds at the test temperature. The minimum torque can be taken as a measure of the viscosity of the masticated rubber. Theoretically, the torque difference (Δ*M*) represents the shear dynamic modulus, which is indirectly related to the total cross-link density of a rubber compound. The total cross-link density is contributed by the sulphide cross-link's and physical cross-link's [22].

Whenever there is excessive mastication, the viscosity registers a sharp decrease. The maximum (*M*_*h*_) and torque difference (Δ*M* = *M*_*h*_ − *M*_*l*_) increase, but minimum torque *M*_*l*_ decreases with the OWT content increase in the NR rubber composites. The Δ*M* could be used as an indirect indication of the cross-link density of the rubber compound [5]. As can be seen, the values of Δ*M* increase continuously with OWT content increase up to 50 phr and then decrease. The *t*_*c*90_ values, cure rate index (CRI), and *t*_*s*2_ values decrease with OWT content increase in NR rubber.

As compared with PSi, the lower values for all rheometric characteristics of the rubber composites filled with OWT were notably reduced in vulcanization, which shows that OWT can more effectively depress the viscosity of rubber and improve the processability during curing (Table 3). The values of minimum torque (*M*_*l*_) reflected the interactions between particles of filler [23]. The larger *M*_*l*_ for PSi showed the stronger interactions between PSi particles which are attributed to the finer size (15–30 nm average diameter) and abundant –OH groups on the surface of the PSi. However, the weaker interactions between OWT particles indicated by the smaller *M*_*l*_ are related to a slightly large size of OWT (300–500 nm average diameter), its electrical neutral surface, and the small density of –OH groups on the OWT particle surface. This property of OWT filler has great advantages in the case of high viscosity rubber, such as some samples of NR, because it is easy to mix and process. The decrease in *t*_*s*2_ is can also improve the productive efficiency, which is good with regard to prophase vulcanizing operation. When OWT filler content increases in NR/OWT rubber composites, optimal cure time (*t*_*c*90_) is shortening, but vulcanization rate is extending.

### 3.3. Cure Kinetics (Activation Energy of Cross-Link and Reversion Process)

The method for calculating the *E*_ac_ and *E*_ar_ is described in our earlier works [21, 24, 25]. During the vulcanization process, sulfur cross-links are formed between the rubber polymer chains (cross-linking), whereas some of the links decay (reversed). Besides intermolecular cross-link (i.e. between two polymer chain), intramolecular type cross-links are also formed. Intermolecular cross-links contribute to the physical properties of the vulcanizate and the sulfur content (poly-, di-, monosulfide cross-links and cyclic and thiol group formation in the case of intramolecular) in the cross-links determines aging characteristics. Thresholds for these two reactions, the activation energy of cross-link and reversion process of cure are the characteristic parameter of the cure properties of a given rubber compounds and can be used as a criteria for energy compatibility of several rubber compound, composing the product.

Reversion tendency was only observed at higher temperatures. For the stability of the system, the *E*_ar_ must be greater than the *E*_ac_, which is determined by the rheometric curve. That is, the curing process, which has lower activation energy, occurs more readily and rapidly because the energy barrier is lower. The prediction of the curing state is usually determined by rheometer, in which the kinetics is described by the torque variation during vulcanization [26–28].

Table 5 shows kinetic parameter and variation of the *E*_ac_, *E*_ar_, and their relation *E*_ar_/*E*_ac_ with variation of OWT content compared to NR/PSi composites. A compound with the smallest possible *E*_ac_ and higher values for *E*_ar_ and their *E*_ar_/*E*_ac_ has a tendency to retain the basic physical and mechanical characteristics. NR composite with 60 phr of OWT has minimum value of *E*_ac_ (5.6 kJ/mol) and maximum value of *E*_ar_/*E*_ac_ relation (14.9) is obtained for NR/OWT (100/50) composites. Further increase of the amount of OWT does not only indicate filler-elastomer interaction, but also filler-filler interaction and even the formation of a three-dimensional filler matrix.

### 3.4. Mechanical Properties

The addition of fillers to polymeric materials leads to improvement in the mechanical properties of the polymer matrix. The reinforcement effect is directly related to the properties of the interphase and depends on the nature of the specific interactions between polymer and reinforcing fillers [29]. The incorporation of filler into elastomers imparts many interesting and useful properties to the particle filled composite material. It is well known that the properties mainly depend on the dispersion condition of filler particles and their principal relevant properties: particle size, surface area, aggregate structure, surface activity, and rubber-filler interactions [30].

The surface activity, a poorly defined term, but widely used in the filler field, can in a chemical sense related to different chemical groups on the surface. In a physical sense, variations in surface energy determine the capacity and energy of adsorption. The surface chemistry of silica has a significant effect only on the vulcanization behavior of filled compounds. Optimal reinforcing power can be achieved only if the filler is well dispersed in the rubber matrix. The chemical or physical interaction between the filler and the rubber is a further important factor in the reinforcing effect [31]. In the case of carbon black the filler-polymer interaction is mainly of physical nature (physisorption) [32]. Interaction between fillers and rubbers has a significant effect on reinforcement properties of a filled rubber. Rubber-rubber interaction mainly occurs when blends of rubber are used in compounds and are considered to be not as significant as filler-rubber and filler-filler interaction.

Filler-rubber interactions are described by the compatibility of the filler with the rubber, while filler-filler interaction is described by the attraction of filler to itself and the ability to form a network. The most important effect of filler-rubber interactions has to do with the occlusion of rubber. The so-called “bound rubber” is trapped between or within aggregates where it is no longer part of the elastically active rubber matrix. As the amount of carbon increased the bound rubber content also increases. Silica has a high dispersive component with a stronger filler-rubber interaction and a weaker filler network. Filler-filler interactions are a primary mechanism in reinforcement, especially at high filler loading. These interactions depend on chemical interactions between the filler particle surfaces (filler-filler, filler-rubber), physical interactions (van der Waals forces, hydrogen bonding), morphology of the filler network, and filler volume fraction [33].

Mechanical properties of nanocomposites generally depend on factors such as filler content, particle size and shape, the degree of adhesion between the filler and the polymer matrix, and the dispersion degree of the filler within the matrix [34].

Tensile strength is a complex function consisting of the nature and type of cross-link's, cross-link densities, and chemical structure of the used rubber. It is well known that if rubber is deformed by an external force, part of the input energy is stored elastically in the chains and is available (released upon crack growth) as a driving force for fracturing. The remaining energy is dissipated through molecular motions by heat, and as such, it is made unavailable to break the chains. At higher cross-linking levels, chain motions become restricted, and the dense network is incapable of dissipating as much energy. This results in a relatively facile brittle fracture at low elongation [35].

As listed in Table 6, the tensile strength of NR/OWT (100/50) rubber composites is close to that of NR/PSi (100/50) rubber composites and exceeds those from PSi in NR composites. As the OWT content increases, the tensile strength decreases to its optimal value and then increase (Table 5). Generally silica particles tend to agglomerate due to formation of hydrogen bond between the surface hydroxyl groups. Higher amount of nanoparticles tends to increase the surface interaction among themselves and thereby enhanced the tendency for agglomeration. At lower loading of SiO_2_, the nanoparticles were well dispersed and thereby increased the surface area for interaction [36]. NR/OWT (100/20) rubber blend has maximum tensile strength values. When the OWT content increases, the values of the elongation at break decrease. The hardness values increase with OWT content increase in rubber composites. The mechanical properties and chemical bonds between phases formed during the networking process of the NR/OWT (100/50) compared to NR/PSi (100/50) rubber composite are similar. The stable hydrogen bonds formed between modifier molecules and the surfaces of OWT reinforced the intensity of interface interactions among the OWT surface, modifying agent molecules, and rubber molecules, and endow the OWT particle with the desired reinforcing effects. The functional group of the side surface combines with the isoprene macromolecule [5].

The elasticity and tensile strength were decreased with OWT content being increased and facilitated the curing up to the optimal integrated properties in a short sulfuration time, which advanced the productive efficiency and saved processing energy [2].

In Table 5 the effect of filler loading on elongation at break is shown. It indicates that elongation at break (%) decreases gradually with increasing filler loading. The reduction of elongation at break is due to stiffening of the polymer matrix by the filler. Further increase in filler loading causes the molecular mobility decrease due to extensive formation of physical bond between the filler particles and the polymer chain that stiffen the matrix [37].

Resilience is the ratio of energy released by the recovery from deformation to that required to produce the deformation. The rebound resilience is enhanced to some extent as the cross-link density rises, and the resilience is related to the flexibility of the molecular chains; the more flexible the molecular chains, the better the resilience [5].

**Figure 2:**
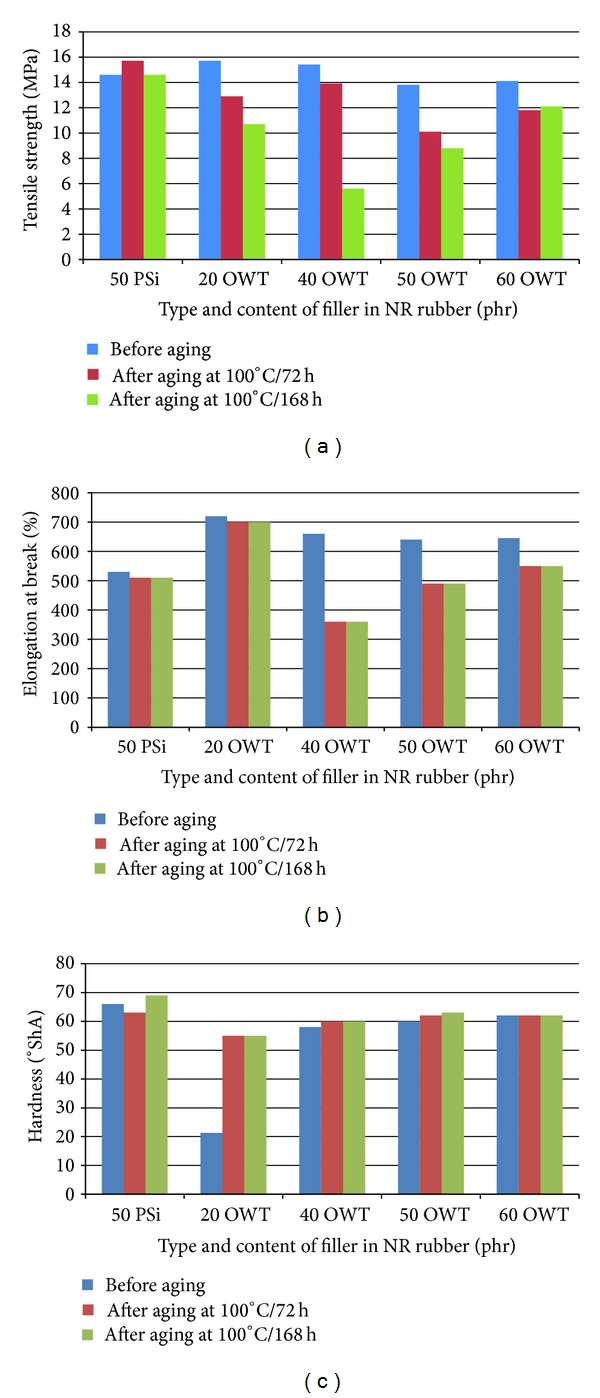
Correlation of mechanical properties: tensile strength (a), elongation at break (b), and hardness (c) on the type and content of filler in NR rubber after aging at 100°C during 72 h and 168 h.

To investigate the influence of thermal aging on the mechanical properties of the rubbers, the cross-linking reactions were performed in an air-circulating oven operated at 100°C for 72 h and 168 h. The retained tensile strength percentage and elongation at break values were then calculated before and after aging. Figures 2(a)–2(c) show the mechanical properties of NR/OWT and NR/PSi rubber composites such as hardness, tensile strength, and elongation at break after thermal aging at 100°C during 72 h and 168 h. The hardness of all rubber blend composites increase with time of aging being increased. This can be attributed to the cross-link density being increased after thermal aging. Well-dispersed nanoparticles resulted in difficulties of heat and mass transfer through the material, thereby preventing fast degradation. Well-dispersed filler acts as a mass transport barrier to oxygen and volatile decomposition products [36]. The tensile strength is a complex function of the nature and type of cross-link's, cross-link densities, the chemical structure of the used elastomers, and the changes associated with degradation. From Figures 2(a) and 2(b), we can observe that there is a marginal decrease in the tensile strength and elongation at break after aging for a period of 72 h. The tensile strength and elongation of NR/OWT rubber composite with 40 phr of filler (sample 3) are reduced by 9.7% and 6.7%, respectively, compared to its original value. These reductions in the properties are due to partial cross-linking of the elastomer backbone and degradation of the rubber taking place upon aging as observed by some of the researchers [38–40]. It needs to be noted that with the increase in temperature, there is more cross-linking of the polymer chain and the filler. The restriction in chain mobility during tensile testing might have also led to the reduction in tensile strength. After 168 h tensile strength and elongation of NR/OWT rubber composite with 60 phr of filler (sample 6) are reduced by 14.2% and 14.7%, respectively, compared to its original value. Values of elongation at break decrease with aging time increase. After thermal aging mechanical properties values decrease and degradation process can be noticed. The changes in the tensile properties upon aging could be due to several reasons such as change in the morphology of the system; degradation of rubber and cross-linking; and change in the level of interaction between components at elevated temperatures. A change in the hardness values increased with increasing OWT loading, which can be attributed to the increased cross-linking density after thermal aging. This can be explained by the sulfur networking addition process of the rubbers and the polysulfide cross-link density reduction process. The polysulfide reacts further to form mono-, di- and cyclic-sulfide bonds during vulcanization via the dissociation, recombination, and rearrangement of the sulfur linkages. There was no change in the mechanical properties value for NR/PSi rubber composites during thermal aging.

### 3.5. Morphology of Rubber Composites

Morphology is a major factor for filler dispersion determined in rubber. It is well known that the phase structure of the rubber is influenced by several factors, including the surface characteristics, filler ratio, viscosity of each component, and compounding process. The primary factor that determines the final morphology of the mixes is their composition. NR with strong molecular polarity has higher surface tension. PSi has a fine particle size and lamellar thickness, uniformity, and limited grain distribution and has excellent surface effects brought about by high specific surface area.

**Figure 3:**
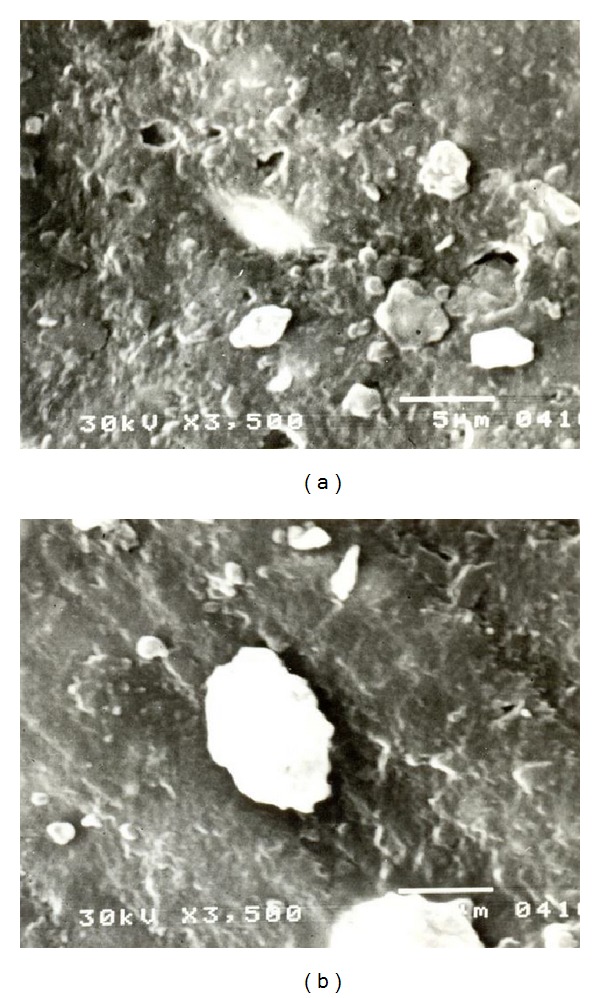
SEM micrographs of NR/OWT (a) and NR/PSi (b) rubber composite with 50 phr of filler.

Figures 3(a) and 3(b) show the morphology of NR composite with 50 phr of OWT and 50 phr of PSi. The dispersion of OWT filler in the rubber is not uniform and its heterogeneous nature is indicative. Two phases with irregular shape can be observed. This means that OWT is poorly dispersed and interphase adhesion between NR and OWT is weak. Thus, free silica particles with relatively more unreacted hydroxyl groups easily promote aggregation through hydrogen bonding [19].

OWT has arranged themselves directionally, parallel in the rubber matrix. The crack extension of the rubber between the parallel OWT sheets will be hindered, passivated, and terminated. The crack cannot penetrate the rubber composites, and the OWT thus yields a good reinforcing effect.

## 4. Conclusions

When OWT filler content increase in NR/OWT rubber composites, optimal cure time (*t*_*c*90_) is shortened, but vulcanization rate is extended which advances the productive efficiency and saves considerable energy. The tensile capability is close to that of rubber filled with precipitated silica, but the tear strength and modulus are inferior to that of PSi rubber composites. In NR/OWT rubber composites filled with 20 and 40 phr of OWT tensile strength are considerably higher than NR/PSi filled with 50 phr of PSi. NR/OWT rubber composites also present excellent elasticity.

NR composite with 60 phr of OWT has minimum value of *E*_ac_ (5.6 kJ/mol) and maximum value of *E*_ar_/*E*_ac_ relation (14.9) and is obtained for NR/OWT (100/50) composites. After thermal aging mechanical properties values decrease and degradation process can be noticed. Morphology study of NR/OWT (100/50) rubber composites shows poorly dispersed OWT in the NR matrix and weak interphase adhesion between NR and OWT as result of the filler particle size (40 *μ*m). OWT as a natural filler can be substitute of PSi in many rubber products with wide potential applied.

## Figures and Tables

**Table 1:** The chemical composition and physical properties of OWT and PSi.

The chemical composition (%)	OWT	PSi	The physical properties	OWT	PSi

SiO_2_	94.27	76	Specific weight (g/cm^3^)	2.27	2
Al_2_O_3_	2.54	7	BET surface (m^2^/g)	39.3	60
Fe_2_O_3_	0.57	—	pH	7	11.5
CaO	0.9	—	Density (kg/m^3^)	370	140
MgO	Trace	—	Sieve residue (%)	0.15	—
Na_2_O	0.06	7			
K_2_O	0.08	—			

**Table 2:** Formulation of the composites based of NR/PS and NR/OWT characteristics.

Sample	Compounds (phr)^a^						
	NR/PSi	NR/OWT	ZnO	Stearic acid	Vulkacit DM^b^	Vulkacit D^b^	Sulfur
1	100/50	—	2.5	1.4	2.5	0.1	1.4
2	—	100/20	2.5	1.4	2.5	0.1	1.4
3	—	100/40	2.5	1.4	2.5	0.1	1.4
4	—	100/50	2.5	1.4	2.5	0.1	1.4
5	—	100/60	2.5	1.4	2.5	0.1	1.4

^a^Parts per hundred; ^b^accelerators Vulkacit DM-2-benzothiazol-2-yldisulfanylbenzothiazole, Vulkacit D-diphenyl guanidine.

**Table 3:** Rheometric characteristics of NR/PSi and NR/OWT rubber composites.

Sample	NR/PSi (phr)	NR/OWT (phr)	*M*_*h*_ (dNm)	*M*_*l*_ (dNm)	ΔM (dNm)	*t*_*s*2_ (s)	*t*_*c*90_ (s)	CRI (s^−1^)
1	100/50	—	5.3	0.9	4.4	60	198	0.72
2	—	100/20	3.4	0.4	3.0	102	316	0.70
3	—	100/40	3.8	0.4	3.4	84	210	0.79
4	—	100/50	3.9	0.4	3.5	84	206	0.82
5	—	100/60	3.8	0.3	3.5	80	214	0.75

**Table 4:** Assignment of the bands in the FTIR spectra of OWT and PSi.

Assignment	OWT	PSi
Si–O out of plane deformation	466 cm^−1^	467 cm^−1^
Si–O bending	792 cm^−1^	795 cm^−1^
Si–OH stretching	907 cm^−1^	—
Si–O–Si stretching	1119 cm^−1^	1088 cm^−1^
O–H (molecular water)	1635 cm^−1^	1641 cm^−1^
OH stretching	3438 cm^−1^	3480 cm^−1^

**Table 5:** Kinetic parameter and values for *E*_ac_ and *E*_ar_ for NR/PSi and NR/OWT rubber composites.

Sample	NR/PSi (phr)	NR/OWT (phr)	*k*_1_(s^−1^)	*k*_2_(s^−1^)	*k*_3_(s^−1^)	*k*_4_(s^−1^)	*E*_ac_ (j mol^−1^)	*E*_ar_ (j mol^−1^)	*E*_ar_ */ E*_ac_
1	100/50	—	2.9 × 10^−2^	1.2 × 10^−2^	4.2 × 10^−2^	1.9 × 10^−3^	6 × 10^4^	8.9 × 10^4^	1.5
2	—	100/20	3.7 × 10^−2^	7.7 × 10^−4^	4.7 × 10^−2^	1.4 × 10^−3^	4.4 × 10^4^	9.9 × 10^4^	2.3
3	—	100/40	3.8 × 10^−2^	6.6 × 10^−4^	5 × 10^−2^	1.5 × 10^−3^	4.8 × 10^4^	14.7 × 10^4^	3.1
4	—	100/50	4.7 × 10^−2^	5.3 × 10^−4^	5.1 × 10^−2^	1.5 × 10^−3^	1.2 × 10^4^	17.9 × 10^4^	14.9
5	—	100/60	3.8 × 10^−2^	8.5 × 10^−4^	5.2 × 10^−2^	1.9 × 10^−3^	5.6 × 10^4^	14.2 × 10^4^	2.5

*k*_1_, *k*_2_ at *T*_1_(180°C).

*k*_3_, *k*_4_ at *T*_2_(190°C).

**Table 6:** Mechanical properties of NR/PS and NR/OWT rubber composites.

Sample	NR/PSi	NR/OWT	Tensile strength (MPa)	Elongation at break (%)	Hardness (°ShA)	Resilience (%)	Tear strength (N/mm)	Modulus at 200% elongation (MPa)	Modulus at 300% elongation (MPa)	Compression set (%) 22 h/100°C
1	100/50	—	14.6	530	66	58	78.9	4.46	6.9	39.3
2	—	100/20	15.7	720	21.3	56	56	2.45	3.4	31
3	—	100/40	15.4	660	58	54	77.1	3.1	4.6	30
4	—	100/50	13.8	640	60	56	79.3	3.2	5.1	30
5	—	100/60	14.1	645	62	50	80.6	3.4	4.4	36.6
